# Efficacy of Narrow Band Imaging System Combined With Magnifying Endoscopy for Differentiating Type IIa Early Gastric Cancer From Adenoma

**DOI:** 10.4021/gr351w

**Published:** 2011-09-20

**Authors:** Hyung Hun Kim, Moo In Park, Jeong Moon Choi, Seun Ja Park, Won Moon

**Affiliations:** a Department of Internal Medicine, Kosin University College of Medicine, Busan, Korea

**Keywords:** Adenoma, Gastric cancer, Narrow band imaging

## Abstract

**Background:**

It is not always possible for endoscopists to differentiate early gastric cancer from adenoma in 0-IIa type neoplasia. The aim of this study was to assess the relationships between images obtained with a narrow band image system combined with magnifying endoscopy (MENBI) and histological findings, especially vascular patterns, to distinguish adenoma from type IIa early gastric cancer (EGC IIa).

**Methods:**

We postoperatively confirmed and evaluated 46 elevated lesions, 32 adenomas and 14 EGC IIa in patients who had undergone endoscopic submucosal dissection. We randomly selected three sites from each neoplasm. The selected sites were classified as four irregular microvascular patterns (IMVPs). In addition, the selected sites were divided into two groups based on the presence of corkscrews.

**Results:**

Regarding IMVP subcategories, (1) slight intrastructural irregular microvascular patterns (ISIMVPs) accounted for 84%, (2) severe ISIMVPs accounted for 6%, (3) fine networks (FNs) accounted for 10%, and (4) corkscrews accounted for 0 of cases in the adenomas. The corresponding proportions in the EGC IIa were (1) 24%, (2) 31%, (3) 45%, and (4) 0. Slight ISIMVPs, severe ISIMVPs, and FNs reliably distinguished the two diseases: P < 0.001 for slight ISMVPs; P < 0.001 for severe ISIMVPs; P < 0.001 for FNs. The presence of corkscrews was observed in 9.5% of EGC IIa and 0 of adenoma cases (P = 0.008).

**Conclusions:**

MENBI can be used to differentiate EGC IIa from gastric adenoma based on IMVPs classifications and the presence of corkscrews.

## Introduction

It is imperative for an endoscopist to make a differential diagnosis between benign and malignant lesions. For such purposes, magnification endoscopy is a powerful tool for correctly diagnosing superficial neoplasm throughout the entire gastrointestinal tract [1-3]. In addition to the minute structure of the mucosa, the microvascular pattern in the cancerous lesion itself is very important for determining a tumor’s malignant potential [4]. The narrow band imaging (NBI) system is an innovative optical technology that can provide clear imaging of the microvascular structure in the mucosal layer. It illuminates the tissue surface through special filters that narrow the red, green, and blue bands with increased intensity of the blue band to enhance the tissue microvascular structure as a result of the differential optical absorption of light by hemoglobin [5]. NBI, in combination with a magnifying endoscope, (MENBI) can yield very clear images of microvessels on mucosal surfaces [6]. According to the Paris classification, superficial gastrointestinal neoplasms can divided into 3 types; superficial elevated (0-IIa) type, superficial flat (0-IIb) type, and superficial depressed (0-IIc) type [7]. Among these three types, it is not always possible for an endoscopist to differentiate early gastric cancer from adenoma in 0-IIa type neoplasms. Because tissue biopsy can only evaluate a tiny piece of the superficial layer of the tumor, it is unable to determine the entire nature of a tumor [8]. Application of endoscopic submucosal dissection (ESD) or endoscopic mucosal resection has made it possible to definitively diagnose many preoperative biopsy-demonstrated adenomas as carcinomas [9, 10]. Changes of diagnosis from adenoma to carcinoma, after ESD, require further investigation and a change in therapeutic strategies. Moreover, in practice, many cases that are diagnosed as adenoma are merely monitored [11]. Therefore, the ability to differentiate between carcinoma and adenoma is crucial in establishing appropriate therapeutic strategies for adenomatous lesions. For providing one answer for this problem, we aimed to evaluate the efficacy of MENBI for differentiating gastric carcinoma from adenoma in superficial elevated (0-IIa) lesions by acquiring detailed MENBI examinations.

## Materials and Methods

### Patients and specimens

This study involved retrospective evaluation using our ESD database. Forty-six neoplasms were obtained from patients who had undergone ESD for 0-IIa type lesions according to the Paris endoscopic classification at the Gospel Hospital, Kosin University College of Medicine, Busan, Korea during the period from May 2009 to December 2010. Of these 46 lesions, 32 and 14 lesions, respectively, were postoperatively confirmed to be adenoma and carcinoma. Lesions diagnosed as mucosal low-grade neoplasia of category 3 according to the revised Vienna classification were defined as gastric adenomas [12]. If a whole part of the resected lesion was pathologically diagnosed as intramucosal carcinoma of category 4.2 according to the revised Vienna classification, the lesion was defined as a gastric carcinoma. Each of the patients gave his or her written informed consent. The Ethical Committee of the Kosin University College of Medicine approved the study.

### Postoperative examination with MENBI

**Figure 1:**
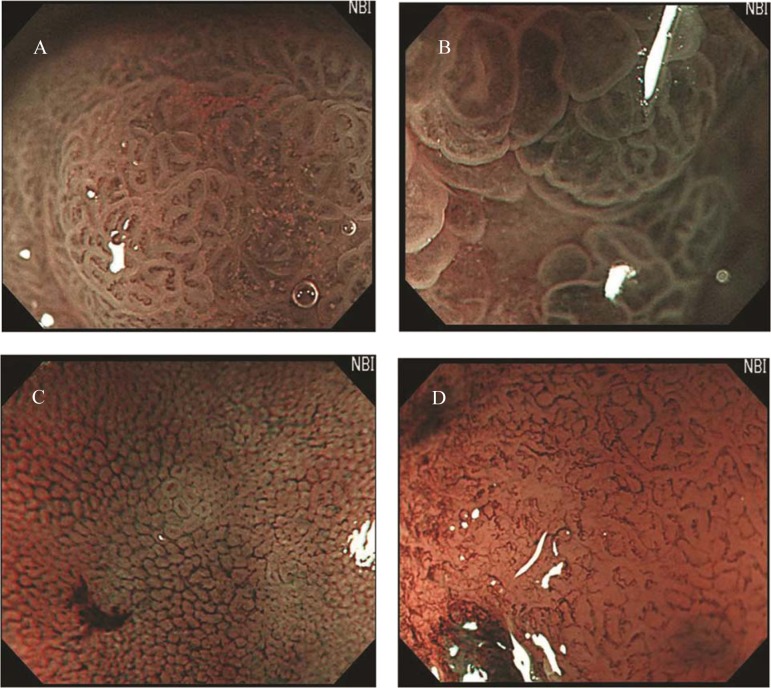
Classification of irregular microvascular patterns observed by MENBI: (A) Slight ISIMVPs, defined by the presence of tiny spiral blood vessels within the fine lobular superficial structure; (B) Severe ISIMVPs, defined by the presence of vertical spiral blood vessels within the coarse lobular superficial structure; (C) Fine networks, defined by fine tubular structures surrounded by a thin microvasculature; (D) Corkscrew patterns, defined by obliterated surface structures and irregular vascular patterns without loop formation. (MENBI: narrow band imaging system in combination with a magnifying endoscope; ISIMVPs: intrastructual irregular microvascular patterns)

**Figure 2:**
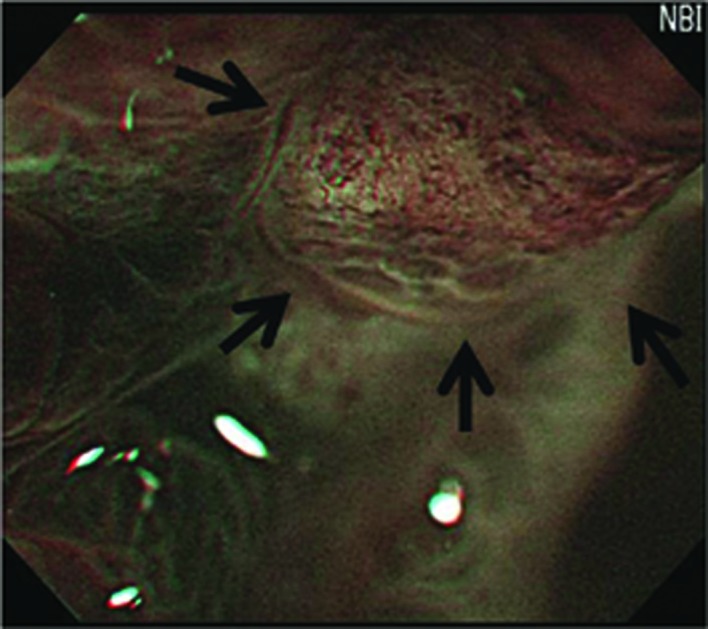
Image of the presence of corkscrews. (The presence of corkscrews occupying over 10% of the observed field is demonstrated (black arrows). Corkscrew was defined by obliterated surface structures and irregular vascular patterns without loop formation)

Preoperative endoscopic examinations were performed by conventional endoscopy and chromoendoscopy. The endoscopic examinations and ESD were performed by a single expert endoscopist. Endoscopy was performed using an upper-GI endoscope (H260Z, Olympus, Tokyo, Japan). The gross findings, such as lesion color, margin, size, and central concavity, of 0-IIa lesions were evaluated by nonzoom endoscopic observation. ESD was performed for all lesions using an insulation tipped diathermic knife (KD-610L, Olympus, Tokyo, Japan) to remove tumors with a minimum lateral margin of 5 mm. Just after removal of the adenomatous lesions, we performed MENBI (GIF-H260Z; Olympus, Tokyo, Japan; CLV 260SL, Olympus, Tokyo, Japan) on fresh specimens. We screened the complete lesion and picked the three sites, presumably highest grade alteration for MENBI. IMVP cases were subcategorized as (1) slight intrastructual irregular microvascular patterns (ISIMVPs), defined by the presence of thin spiral blood vessels within the fine lobular superficial structure, (2) severe ISIMVPs, defined by the presence of vertical spiral blood vessels within the coarse lobular superficial structure, (3) fine networks, defined by fine tubular structures surrounded by thin microvasculature, or (4) corkscrew patterns, which appear as obliterated surface structures and irregular vascular patterns without loop formation (Fig. 1). Adding to these classifications, we evaluated the presence of corkscrew vascular patterns. The presence of corkscrews was defined by the typical presence of corkscrew vascular patterns in at least over 10% of the observed field (Fig. 2). The resected tumors were measured and fixed with 10% buffered formalin. The removed specimens were sectioned at 2 mm intervals, embedded in paraffin blocks, cut into 5 mm sections, and stained with hematoxylin and eosin for pathologic diagnosis.

### Statistical analysis

Statistical analysis was performed using the Statistical Package for the Social Sciences (SPSS) software (version 16.0, SPSS, Chicago, IL, USA). The relationship between IMVP and pathologic diagnosis was evaluated using the 2-sided Fisher exact test. The differences between GA and EGC IIa were assessed by 2-sided Fisher exact test and Student t-test. Diagnostic efficacy was assessed by specificity and sensitivity. Two-tailed P values less than 0.05 were regarded as statistically significant.

## Results

All lesions were removed by ESD en bloc with a full margin distance to enable subsequent accurate pathologic diagnosis. The average tumor diameter was 1.96 cm in the adenoma group (n = 32) and 2.02 cm in the carcinoma group (n = 14). The majority of lesions in both groups were discolored, although reddish lesions were seen in 9% of cases in the adenoma group and in 14% in the carcinoma group. A central concavity was found in 13% of the adenoma group cases and in 86% of the carcinoma cases (P < 0.001; Table 1).There were no differences in tumor size, lesion color, or the characteristics of margin between the adenoma group and the carcinoma group: P = 0.082, P = 0.633, and P = 0.521 respectively; Table 1).

**Table 1:** Gross Appearance of 0-IIa Lesions

	Adenoma (n = 32)	EGC IIa (n = 14)	P value
Size, mean ± SD, cm	1.96 ± 0.80	2.02 ± 0.53	0.892
Reddish color, n (%)	3 (9)	2 (14)	0.633
Obscure margin, n (%)	1 (3)	1 (7)	0.521
Central concavity, n (%)*	4 (13)	12 (86)	< 0.001

EGC IIa: type IIa early gastric cancer.

All analyses were performed by the 2-sided Fisher’s exact test except tumor size (Student t-test).

* Sensitivity 85.7%, specificity 87.5%, and positive predictive value 75.0% (for EGC IIa).

Regarding IMVP subcategories observed by MENBI, by proportions of lesions, (1) slight ISIMVPs accounted for 84% (81 out of 96), (b) severe ISIMVPs accounted for 6% (6 out of 96), (3) fine networks accounted for 10% (9 out of 96), and (4) corkscrews accounted for 0 (0 out of 96) of cases in the adenoma group. The corresponding proportions in the carcinoma group were (1) 24% (10 out of 42), (2) 31% (13 out of 42), (3) 45% (19 out of 42), and (4) 0 (0 out of 42). Slight ISIMVPs were significant findings for the adenoma group (P < 0.001, sensitivity 84.3%, specificity 76.2%, and positive predictive value 89.0%), and severe ISIMVPs and fine networks were significant findings for the EGC IIa group (P < 0.001, sensitivity 30.9%, specificity 93.5%, and positive predictive value 68.4% for severe ISIMVPs; P < 0.001, sensitivity 45.2%, specificity 90.6%, and positive predictive value 67.8% for severe fine networks; Table 2). The presence of corkscrews was observed more frequently in the EGC IIa group (P = 0.008, sensitivity 9.5%, specificity 100%, and positive predictive value 100%; Table 3), and was correlated with severe ISIMVPs (Table 4).

**Table 2:** Irregular Microvascular Patterns in 0-IIa Lesions

IMVPs	Adenoma (n = 96)	EGC IIa (n = 42)	P value
Slight ISIMVPs*, n (%)	81 (84)	10 (24)	< 0.001
Severe ISIMVPs†, n (%)	6 (6)	13 (31)	< 0.001
Fine network‡, n (%)	9 (10)	19 (45)	< 0.001
Corkscrews, n (%)	0 (0)	0 (0)	N.A

IMVPs: irregular microvascular patterns; ISIMVPs: intrastructual irregular microvascular patterns; EGC IIa: type IIa early gastric cancer; N.A: not available. All analyses were performed by the 2-sided Fisher’s exact test.

* Sensitivity 84.3%, specificity 76.2%, and positive predictive value 89.0% (for adenoma).

† Sensitivity 30.9%, specificity 93.5%, and positive predictive value 68.4% (for EGC IIa).

‡ Sensitivity 45.2%, specificity 90.6%, and positive predictive value 67.8% (for EGC IIa).

**Table 3:** The Presence of Corkscrews in 0-IIa Lesions

The presence of corkscrews	Adenoma (n = 96)	EGC IIa (n = 42)	P value
Positive, n (%)*	0 (0)	4 (9.5)	0.008
Negative, n (%)	96 (100)	38 (88)	

EGC IIa: type IIa early gastric cancer.

The definition of the presence of corkscrew is the observation of corkscrew vascular changes in over 25% of a selected field.

All analyses were performed by the 2-sided Fisher’s exact test.

* Sensitivity 9.5%, specificity 100%, and positive predictive value 100% (for EGC IIa).

**Table 4:** The Presence of Corkscrews and IMVPs

IMVPs	The presence of corkscrews		P value
	Positive	Negative	
Slight ISIMVPs, n = 91 (%)	0 (0)	91 (100)	0.012
Severe ISIMVPs, n = 19 (%)	4 (21)	15 (79)	< 0.001
Fine network, n = 28 (%)	0 (0)	28 (0)	0.582

IMVPs: irregular microvascular patterns; ISIMVPs: intrastructual irregular microvascular patterns; EGC IIa: type IIa early gastric cancer.

All analyses were performed by the 2-sided Fisher’s exact test.

## Discussion

The normal patterns of the surface pits and microcirculation are altered in cancerous mucosa [13, 14]. Magnifying endoscopic images, with regard to the minute structure, corresponded well with the histopathological results and emphasized the importance of the tumor’s microvascular pattern in addition to the minute structure [15]. The NBI system is a specialized tool for visualizing mucosal microvascular patterns with a narrow band wave length. One of the greatest advantages of MENBI is that it enhances visualization of the mucosal structures and the microvascular patterns thereby providing a more detailed assessment of characteristics of a neoplasm than any other practical modalities. Indeed, in the present study, MENBI-enhanced magnified images of the lesions enable clear visualization of IMVPs. This observation is consistent with the results of other MENBI studies of early stage gastric cancer [6, 16].

According to 3 previous studies on the differential diagnosis of gastric carcinoma from adenomatous lesions using MENBI, the investigation of both superficial structure and vascular patterns is useful for the differential diagnosis of adenoma and carcinoma [6, 16, 17]. Nakamura et al analyzed 14 cases and randomly selected 5 points from each case to compensate for the relatively small number of samples [16]. This study showed that slight ISIMVPs were important findings specific to adenoma, and severe ISIMVPs or fine network patterns were highly specific to EGC IIa [16]. These findings fairly coincide with our results (Table 2). While Nakamura et al used special method, 5 sites per lesion for MENBI, to compensate the limitations of small numbers of cases, the small sample size cannot but predicate the lack of statistical significance. Moreover, their method neglected the advantage of endoscopy over histology: the ability to be able to screen the complete lesion and pick the presumably highest grade alteration for evaluation. Our larger scale investigation based on 46 cases with random selection of 3 points, presumably highest grade alteration, from each 0-IIa lesion strongly supports the finding of the previous study that MENBI can be used to differentiate EGC IIa from adenoma based on IMVPs.

Apart from the classical categories of IMVPs defined by Nakamura et al, we also assessed the presence of corkscrews and revealed that the presence of corkscrews can be also helpful to draw a sharp line between EGC IIa and adenoma (P = 0.008; Table 3). Moreover, the presence of corkscrews was only observed with severe ISIMVPs in the EGC IIa group. Corkscrews were not observed in 0-IIa lesions, but were often found in 0-IIc or 0-IIb lesions. This may suggest that severe ISIMVPs are related to more advanced vascular changes than fine networks in relation with carcinoma (Table 4).

In gross findings, it was highly suggested that central concavities may help distinguish between adenoma and EGC IIa (Table 1). However, reddish lesions and obscure margin, thought to indicate EGC IIa [18] were neither specific for EGC IIa nor adenoma.

The use of ESD for treating adenoma as well as early gastric cancer has currently been popularized with rapid technical progress. Postoperative pathologic evaluation revealed that a few 0-IIa lesions, diagnosed as adenoma by preoperative forcep biopsies, contain carcinomatous components. Adenomatous lesions have often been observed due to the idea that the malignant potential is relatively low from a long-term report [18]. However, just following up on 0-IIa adenomatous lesions that have carcinomatous foci can make it possible to miss the right time for ESD. This is the reason why accurate diagnosis is crucial for 0-IIa lesions. In this light, our result can be applied not only to acquire an accurate impression by observing IMVPs, but also to perform precise target biopsies from potential cancerous portions on 0-IIa lesions.

In conclusion, MENBI can be used as a highly effective tool to differentiate EGC IIa from gastric adenoma based on IMVP classification and the presence of corkscrews in 0-IIa lesions that are difficult to distinguish by conventional endoscopy. Moreover, it can also be used for precise target biopsies to avoid missing cancerous components. Severe ISIMVPs may reflect more advanced vascular changes than fine networks and slight ISIMVPs. This theory needs to be verified by pathologic evaluation with MENBI examination in the future.
